# Sodium–Glucose Cotransporter 2 Inhibitors Work as a “Regulator” of Autophagic Activity in Overnutrition Diseases

**DOI:** 10.3389/fphar.2021.761842

**Published:** 2021-10-21

**Authors:** Kazuhiko Fukushima, Shinji Kitamura, Kenji Tsuji, Jun Wada

**Affiliations:** Department of Nephrology, Rheumatology, Endocrinology and Metabolism, Okayama University Academic Field of Medicine, Dentistry and Pharmaceutical Sciences, Okayama, Japan

**Keywords:** SGLT2 inhibitor, autophagy, diabetic kidney disease, mTOR, SIRT1, HIFs, secretory autophagy, autophagic cell death

## Abstract

Several large clinical trials have shown renal and cardioprotective effects of sodium–glucose cotransporter 2 (SGLT2) inhibitors in diabetes patients, and the protective mechanisms need to be elucidated. There have been accumulating studies which report that SGLT2 inhibitors ameliorate autophagy deficiency of multiple organs. In overnutrition diseases, SGLT2 inhibitors affect the autophagy *via* various signaling pathways, including mammalian target of rapamycin (mTOR), sirtuin 1 (SIRT1), and hypoxia-inducible factor (HIF) pathways. Recently, it turned out that not only stagnation but also overactivation of autophagy causes cellular damages, indicating that therapeutic interventions which simply enhance or stagnate autophagy activity might be a “double-edged sword” in some situations. A small number of studies suggest that SGLT2 inhibitors not only activate but also suppress the autophagy flux depending on the situation, indicating that SGLT2 inhibitors can “regulate” autophagic activity and help achieve the appropriate autophagy flux in each organ. Considering the complicated control and bilateral characteristics of autophagy, the potential of SGLT2 inhibitors as the regulator of autophagic activity would be beneficial in the treatment of autophagy deficiency.

## Introduction

The sodium–glucose cotransporter 2 (SGLT2) inhibitor is an antidiabetic drug which lowers the blood glucose level by increasing the excretion of glycosuria. Several large clinical trials have shown the renal and cardioprotective effects of SGLT2 inhibitors in diabetes patients, and some of those effects are independent of the improvement of the blood glucose level (Zinman et al., 2015; Wanner et al., 2016; Perkovic et al., 2019). A huge amount of research has been conducted to elucidate the mechanisms of those protective effects of SGLT2 inhibitors. SGLT2 is expressed on the apical side of proximal tubular cells and cotransports one Na^+^ ion and one molecule of glucose. In diabetes, SGLT2 is overexpressed and the increasing amount of Na^+^ and glucose is reabsorbed (Wang et al., 2017). SGLT2 inhibitors suppress the reabsorption of Na^+^ and glucose; the former induces mainly hemodynamic changes and the latter induces metabolic changes, respectively. Suppression of Na^+^ reabsorption increases the amount of Na^+^ and Cl^−^ reaching the macula densa, followed by constriction of afferent arteriole and mitigation of glomerular hyperfiltration (tubuloglomerular feedback) in diabetic kidney disease (DKD) (Cherney et al., 2014; Kidokoro et al., 2019). The SGLT2 inhibitor is also reported to improve glomerular hyperfiltration of DKD *via* dilation of the efferent arteriole (van Bommel et al., 2020). It also increases Na^+^ excretion into urine, which reduces cardiac filling pressures and causes neurohormonal changes, resulting in cardioprotective effects (Packer, 2020c). On the other hand, suppression of glucose reabsorption improves lipid metabolism, resulting in the reduction of oxidative stress, inflammasome, and amelioration of mitochondrial dysfunction (Kamezaki et al., 2018; Takagi et al., 2018; Tanaka et al., 2018). It enhances free fatty acid oxidation and increases the production of ketone bodies, inducing renal and cardioprotective effects in diabetes (Cowie and Fisher, 2020; Tomita et al., 2020). Thus, hemodynamic and metabolic changes by SGLT2 inhibitors are considered to induce renal and cardioprotective effects in diabetes.

Recently, autophagy deficiency is reported to cause organ damages in overnutrition diseases such as type 2 diabetes and obesity (Yamamoto et al., 2017; Takagaki et al., 2020). Autophagy is a lysosome-dependent degradation mechanism of the cell, which digests intracellular components in starvation or degrades dysfunctional organella, playing an important role in maintaining the cellular homeostasis. In overnutrition diseases, stagnation of autophagy causes accumulations of damaged organella and denatured proteins, resulting in cellular and organ damages (Yamamoto et al., 2017; Fukushima et al., 2020). In many lines of evidence, therapeutic targeting of autophagy has been attracting much attention in overnutrition diseases, but there are at least two difficulties in therapeutically regulating autophagic activity. First, autophagy activity is controlled by various factors. Autophagy consists of multiple steps: sequestration of cytosolic components by autophagosome, autolysosome formation by the fusion of autophagosome and lysosome, and degradation of intracellular components in autolysosome, and each step is controlled by various signaling pathways. Therefore, therapeutic targeting of autophagy needs the systematic regulation of signaling which affects the autophagy activity. Second, the “appropriate” degree of autophagy activity is different depending on organs, diseases, or situations. Recently, it turned out that not only stagnation but also overactivation of autophagy causes cellular damages. Rubicon, a negative regulator of autophagy, is downregulated in adipocytes and autophagy is overactivated while it is upregulated in hepatocytes and autophagy is stagnated in aged mice, and both cells are damaged by their autophagy deficiencies (Yamamuro et al., 2020). In addition, in the mouse model of myocardial ischemia–reperfusion (I/R) injury, excessive autophagy is reported to cause the autophagic cell death of myocardial cells in the reperfusion phase (Liu et al., 2013). Therefore, therapeutic interventions which simply enhance or stagnate autophagy activity might be a “double-edged sword” in some situations. Thus, therapeutic targeting of autophagy seems difficult because of the complicated control and bilateral characteristics of autophagy.

Recently, there have been accumulating studies which report that SGLT2 inhibitors ameliorate autophagy deficiency of multiple organs such as the kidney and the heart. Most of the studies are based on the overnutrition disease models, but some studies are based on non-overnutrition diseases. In this article, we review how and in which organs SGLT2 inhibitors improve autophagy deficiency, and we also mention some existing medicines which are reported to improve autophagy.

## Autophagy-Controlling Signaling Pathways in Overnutrition Diseases and the Effect of Sodium–Glucose Cotransporter 2 Inhibitors on Them

**FIGURE 1:**
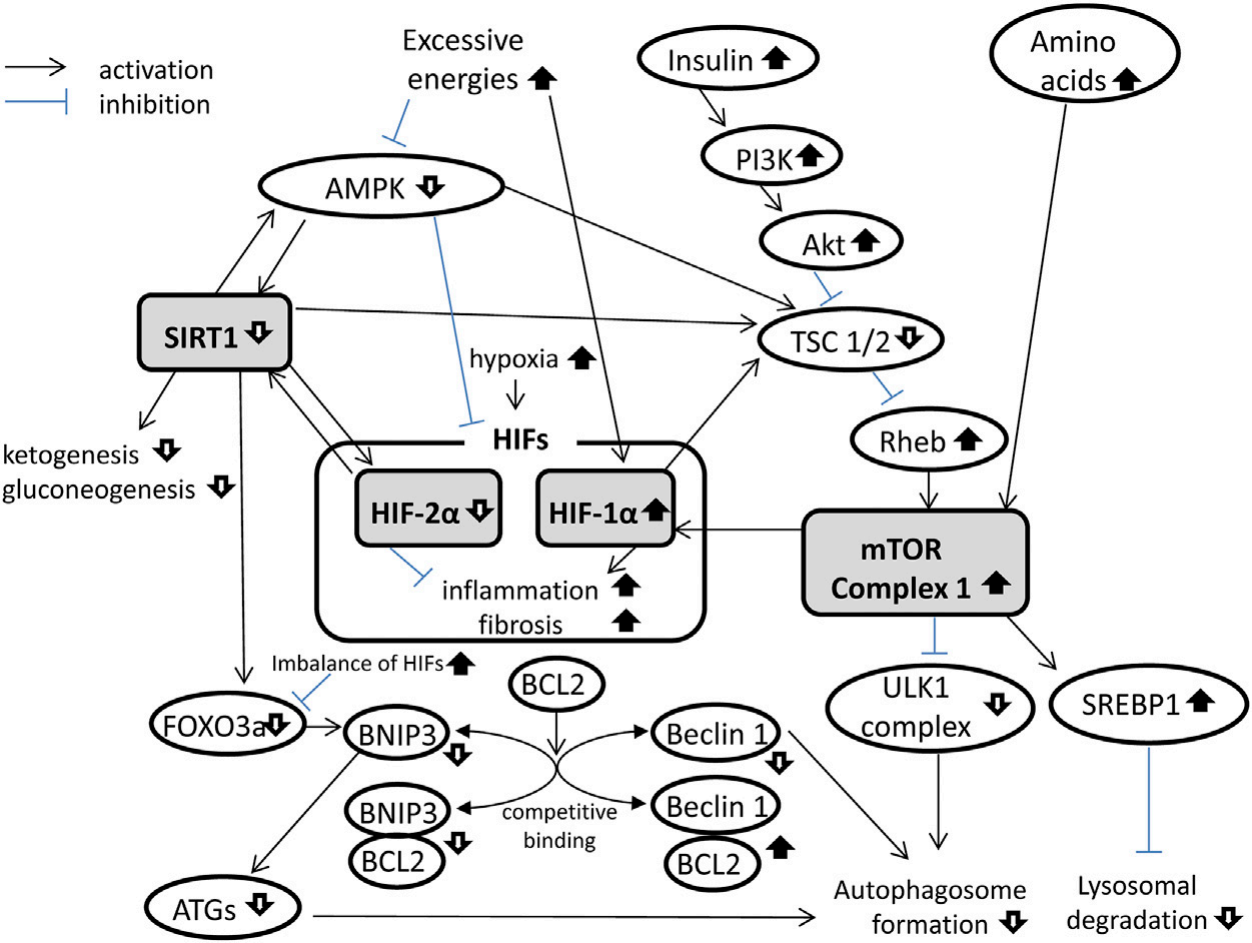
Mammalian target of rapamycin (mTOR), sirtuin 1 (SIRT1), and hypoxia-inducible factor (HIF) signaling pathways in overnutrition diseases. mTOR complex 1 (mTORC1) formation is activated, whereas SIRT1 is suppressed, and HIFs are imbalanced in overnutrition diseases. These signaling pathways interact with each other and suppress the autophagy flux. AMPK, AMP-activated protein kinase; ATGs, autophagy-related genes; BCL2, B-cell lymphoma 2; BNIP3, BCL2-interacting protein 3; FOXO3a, forkhead box O3a; PI3K, phosphatidylinositol-3 kinase; Rheb, Ras homolog enriched in brain; SREBP1, sterol regulatory element–binding protein 1; TSC, tuberous sclerosis complex; ULK1, Unc-51–like autophagy-activating kinase 1.

Autophagy flux deficiency causes multiple organ injuries in overnutrition diseases such as type 2 diabetes mellitus and obesity. In recent studies researching the organ protective mechanisms of SGLT2 inhibitors in overnutrition diseases, SGLT2 inhibitors are shown to ameliorate autophagy deficiency. Inhibition of SGLT2 induces fasting-like and hypoxia-like transcriptional changes (Osataphan et al., 2019), which activate autophagy in overnutrition diseases. At least three signaling pathways are involved in SGLT2 inhibitor’s effect on autophagy flux in overnutrition diseases: mammalian target of rapamycin (mTOR), sirtuin 1 (SIRT1), and hypoxia-inducible factors (HIFs) pathways. These three pathways are affected by fasting-like state induced by SGLT2 inhibitors and interact with each other (Figure 1).

### Mammalian Target of Rapamycin Pathway

mTOR is a serine–threonine kinase that senses nutritional status of the cellular environment and regulates proliferation, growth, metabolism, and anabolism of the cell (Jewell and Guan, 2013). mTOR is activated by glucose, amino acids, insulin, and/or insulin-like growth factor and then forms mTOR complex 1 (mTORC1). mTORC1 enhances anabolism (synthesis of protein and lipid) and suppresses catabolism including autophagy. mTORC1 binds to Unc-51–like autophagy-activating kinase 1 (ULK1) and inhibits the ULK1 complex, which consists of ULK1, mATG13, and FIP200, and is essential for autophagosome formation (Mizushima, 2010). In overnutrition diseases, overactivated mTORC1 inhibits autophagosome formation. Moreover, mTORC1 enhances sterol regulatory element–binding protein (SREBP) 1 (Li et al., 2010), increases lipid deposition in lysosome, and decreases degradation capacity of lysosome (Rampanelli et al., 2018), which also interferes with autophagy flux. Thus, the mTOR pathway suppresses autophagy flux in overnutrition diseases and, in fact, inhibition of mTORC1 by rapamycin ameliorates autophagy deficiency (Su et al., 2018) and improves DKD (Yang et al., 2007; Mori et al., 2009).

SGLT2 inhibitor suppresses those factors which induce mTORC1 formation. Inhibition of SGLT2 increases glucose excretion in urine, induces loss of calories, and then activates AMP kinase (AMPK), resulting in the mTORC1 suppression. Glucose excretion in urine by SGLT2 inhibitors decreases not only serum glucose but also serum insulin (Kitada et al., 2017; Sugizaki et al., 2017), which suppresses phosphatidylinositol-3 kinase (PI3K) activation. PI3K suppression induces inhibition of phosphoinositide-dependent kinase (PDK) 1 and Akt (protein kinase B), activation of the tuberous sclerosis complex (TSC) 1/2, and inhibition of Ras homolog enriched in brain (Rheb), resulting in mTORC1 inhibition (Dibble and Cantley, 2015). In addition, an increase in the glucagon/insulin ratio by SGLT2 inhibitors activates gluconeogenesis in the liver, consuming the circulating amino acids, which also suppresses mTORC1 and activates autophagy (Esterline et al., 2018). Thus, SGLT2 inhibitor suppresses mTORC1 formation by decreasing serum glucose and insulin and then improves autophagy flux in overnutrition diseases.

### Sirtuin 1 Pathway

SIRT1 is a nicotinamide adenine dinucleotide (NAD)–responsive deacetylase and is one of sensors being activated in response to nutrient deprivation. Especially, SIRT1 is called the “ketogenic starvation sensor,” for SIRT1 promotes fatty acid oxidation and gluconeogenesis and inhibits glycolysis, resulting in ketogenesis promotion (Gillum et al., 2011). In starvation, AMPK activation increases intracellular NAD^+^ production, resulting in SIRT1 activation (Canto et al., 2009). SIRT1 also activates AMPK *via* deacetylation of LKB1 (Price et al., 2012), so SIRT1 and AMPK stimulate each other in the setting of starvation. Besides promotion of fatty acid oxidation, SIRT1 induces many changes which mitigate organ injuries in overnutrition diseases: mitochondrial biogenesis (Tang, 2016), antioxidative stress, and, above all, autophagy (Iside et al., 2020). For example, SIRT1 enhances the selective autophagy of damaged mitochondria (Fang et al., 2014). SIRT1 deacetylates FOXO3a, which activates BNIP3, resulting in upregulation of autophagy-related proteins such as LC3, ATG5, and ATG7 (Kume et al., 2010; Lee, 2019). In addition, SIRT1 suppresses mTORC1 formation *via* activating TSC2 (Ghosh et al., 2010) and activates HIF-2α (Chen et al., 2012), which could also enhance autophagy flux.

In overnutrition diseases, SGLT2 is upregulated by environmental glucose, which promotes energy uptake, lipid synthesis, and storage, resulting in SIRT1 suppression in proximal tubules (Umino et al., 2018). In addition, serum SIRT1 levels are decreased in patients with type 2 diabetes (Packer, 2020e). Pharmacological inhibition of SGLT2 induces SIRT1 upregulation in proximal tubules (Umino et al., 2018). Besides proximal tubular cells, SGLT2 inhibitors have been shown to upregulate SIRT1 of other cells which do not express SGLT2, for example, cardiac and hepatic cells (Packer, 2020d). The systemic upregulation of SIRT1 is considered to occur owing to AMPK activation and NAD^+^ production as a result of energy deprivation by SGLT2 inhibitors (Packer, 2020d). Thus, SGLT2 inhibitor upregulates SIRT1 and enhances autophagy flux in overnutrition diseases.

### Hypoxia-Inducible Factor Pathway

HIFs are transcription factors that respond to hypoxia. In normal circumstances, HIF-α is constantly synthesized but degraded by prolyl hydroxylases (PHDs), so intracellular concentration of HIF-α is kept low (Hirota, 2021). Under hypoxic conditions, PHDs are inactivated and HIF-α translocates into the nucleus, binds to HIF-1β, and then forms heterodimer (Hirota, 2021). The heterodimer of HIFs then binds to the hypoxia-responsive element (HRE) region of the DNA, resulting in the transcription of genes that are involved in the response to hypoxia conditions (Hirota, 2021). The HIF-α family consists of HIF-1α, HIF-2α, and HIF-3α. HIF-1α mainly activates the transcription of cytosolic and mitochondrial proteins which decrease oxygen consumption and synthesis of reactive oxygen species (ROS) (Schonenberger and Kovacs, 2015). HIF-2α primarily enhances erythropoietin synthesis and iron metabolism (Eckardt and Kurtz, 2005). The HIF-3α gene is considered to target the HIF-1 gene and modulates hypoxic gene induction (Tanaka et al., 2009).

HIFs play an important role in enhancing autophagy. In fact, autophagy is activated in hypoxia conditions (Bellot et al., 2009), and HIF-activating medicine, such as cobalt, also promotes autophagy (Chen et al., 2017). HIF-1α and HIF-2α promote autophagy *via* activating BNIP3, followed by Beclin1 activation (Bellot et al., 2009; Zhu et al., 2019). HIF-1α also enhances autophagy *via* activation of TSC 1/2, followed by suppression of Ras homolog enriched in brain (Rheb) and inhibition of mTORC1 formation (Brugarolas et al., 2004). On the other hand, HIF-2α promotes autophagy *via* SIRT1 activation (Chen et al., 2011), so SIRT1 and HIF-2α stimulate and reinforce each other. HIF-1α promotes the autophagy of damaged mitochondria (Schonenberger and Kovacs, 2015), and HIF-2α promotes that of dysfunctional peroxisomes (Walter et al., 2014). Although prolonged activation of HIF-1α causes inflammation and fibrosis (Luo et al., 2015), that was ameliorated by HIF-2α activation (Ban et al., 2014; Kong et al., 2017). Therefore, the balance and cooperation of HIF-1α and HIF-2α are important for autophagy without inflammation or fibrosis by HIF-1α overactivation (Packer, 2020e). In the kidney, for example, HIF-1α is expressed mainly in the epithelial tubular cells, and HIF-2α is expressed in the interstitial cells and endothelial cells (Rosenberger et al., 2002), and their balance in the kidney is important for renal autophagy without inflammation or fibrosis (Packer, 2020e). In the cells which express both HIF-1α and HIF-2α such as cardiomyocytes and hepatocytes (Jurgensen et al., 2004; Rankin et al., 2007), their intracellular balance and cooperation are also important in the clearance of damaged organelles by autophagy (Packer, 2020e).

In overnutrition diseases, there occurs the imbalance of HIF-1α and HIF-2α; HIF-1α is overactivated (Isoe et al., 2010; Bondeva et al., 2013), while HIF-2α is downregulated (Mitra et al., 2008; Zeng et al., 2015). Overactivation of HIF-1α is induced by high glucose, advanced glycation end products (AGEs), mTOR, and hypoxia (Isoe et al., 2010; Bondeva et al., 2013), whereas downregulation of HIF-2α is induced by SIRT1 suppression in diabetes (Mitra et al., 2008; Zeng et al., 2015). In DKD, HIF-1α overactivation causes fibrosis and HIF-2α downregulation causes renal anemia (Packer, 2020e). SGLT2 inhibitors are reported to improve the imbalance of HIF-1α and HIF-2α. The overactivation of HIF-1α is improved *via* the decrease in serum glucose, AGEs, mTORC1 formation, and the increase of AMPK (Bessho et al., 2019; Kim et al., 2019), and the downregulation of HIF-2α is improved *via* SIRT1 activation (Chen et al., 2011; Chen et al., 2012) by the therapeutic effect of SGLT2 inhibitors. Thus, SGLT2 inhibitors improve the imbalance of HIF-1α and HIF-2α, ameliorate fibrosis and anemia, and contribute to the autophagy deficiency in DKD.

## Effect of Sodium–Glucose Cotransporter 2 Inhibitors on Autophagy in Each Organ

As SGLT2 inhibitor’s protective effect of multiple organs has been revealed in overnutrition diseases, many researchers have focused on the effect of SGLT2 inhibitors on autophagy. At present, many studies have been reported to demonstrate SGLT2 inhibitor’s therapeutic effect on autophagy deficiency in overnutrition diseases (Table 1). The kidney and the heart are well-researched in those studies maybe because of the abundance of preceding clinical and basic studies on those two organs. Interestingly, SGLT2 inhibitors also ameliorated autophagy deficiency of diseases other than overnutrition, such as ischemia and inflammation (Table 1). This indicates that SGLT2 inhibitors improve the autophagy deficiency of diseases which have the same pathway as overnutrition diseases. In fact, SGLT2 inhibitors are expected and being studied whether to ameliorate Alzheimer’s disease and autosomal dominant polycystic kidney disease (ADPKD) *via* improving their autophagy deficiencies, in which diseases the AMPK/mTOR pathway influences their progression. (Esterline et al., 2020; Nowak and Hopp, 2020). When discussing the therapeutic effect of SGLT2 inhibitors on autophagy deficiency, it is important to consider the role of autophagy and what signaling the autophagy flux interacts with in each organ.

**TABLE 1:** Proposed effects of SGLT2 inhibitors on autophagy deficiency in each organ/cell and disease.

Organ/cell	Disease/stress	Proposed mechanisms
Kidney		
Proximal tubular cell	Obesity	AMPK/mTOR signaling (Fukushima et al., 2020)
		Improvement of lysosomal capacity (Fukushima et al., 2020)
	DKD	AMPK/mTOR signaling (Lee et al., 2019)
Podocyte	DKD	Beclin1 activation (Korbut et al., 2020)
(No mention on cell)	Obesity	AMPK/mTOR signaling (Jaikumkao et al., 2021)
Heart	Diabetic hearts	Cardiotoxic lipids reduction and AMPK activation (Aragon-Herrera et al., 2019)
		Inhibiting microRNA-30d expression (Zhang et al., 2020)
	DOX cardiomyopathy	Beclin1/toll-like receptor 9/SIRT3 axis (Wang et al., 2020)
	SNT-induced cardiac dysfunction	AMPK–mTOR signaling pathway (Ren et al., 2021)
	Myocardial infarction	Beclin1 suppression *via* NHE1 inhibition (Jiang et al., 2021)^a^
	Myocardial infarction of diabetic hearts	Increase of BNIP3 expression (Mizuno et al., 2018)
Liver		
Hepatocyte	Hepatic steatosis in type 2 DM	AMPK/mTOR signaling and Beclin1 activation (Li et al., 2021)
	NAFLD	AMPK/mTOR signaling and suppression of SREBP1c (Nasiri-Ansari et al., 2021)
Hepatic macrophage	NAFLD	AMPK/mTOR signaling (Meng et al., 2021)
Colon	Inflammatory bowel disease	AMPK/mTOR signaling pathway and Beclin1 activation (Arab et al., 2021)
Nerve system striatum	Huntington’s disease	Suppression of glycolysis, apoptosis, and inflammation (El-Sahar et al., 2020)
Immune cells	Lipopolysaccharide induction	Inhibiting intracellular glucose metabolism resulting in AMPK activation and p62 increase (Xu et al., 2018)
HepG2 cell	DOX induction	Promoting ULK1 serine 757 phosphorylation (Zhong et al., 2020)^a^

Proposed effects of SGLT2 inhibitors on autophagy deficiency in each organ/cell and disease. AMPK, AMP-activated protein kinase; BNIP3, BCL2-interacting protein 3; DKD, diabetic kidney disease; DM, diabetes mellitus; DOX, doxorubicin; mTOR, mammalian target of rapamycin; NAFLD, nonalcoholic fatty liver disease; NHE1, Na^+^/H^+^ exchanger 1; SIRT3, sirtuin 3; SNT, sunitinib; SREBP1c, sterol regulatory element–binding protein 1c; ULK1, Unc-51–like autophagy-activating kinase 1.

a Indicates the suppression effects of overactivated autophagy, and all the others are the activation effects of stagnated autophagy.

### Kidney

The kidney is one of the organs that are well-studied about the SGLT2 inhibitor’s therapeutic effects. SGLT2 inhibitors ameliorate DKD *via* hemodynamic and metabolic mechanisms such as improvement of tubuloglomerular feedback (Cherney et al., 2014; Kidokoro et al., 2019) and the suppression of claudin-1 in podocytes as a result of SIRT1 upregulation (Hasegawa et al., 2013). These various therapeutic mechanisms of SGLT2 inhibitors would be beneficial to slow the progression of DKD as several pathophysiological disorders contribute to the progression of the disease; therefore, combination of multiple drugs has been prescribed in clinic so far (DeFronzo et al., 2021). The mechanisms of SGLT2 inhibitor’s influence on the kidney is more complicated than those on other organs because of its structure. In other organs, such as the heart or the liver, the majority of cellular population is occupied by a single type of cell and the structure of their tissues are comparably simple. On the other hand, the kidney consists of various types of cells, such as podocytes, mesangial cells, and tubular cells, and those cells form complicated structures of the glomerulus and nephron. Moreover, proximal tubular cells upregulate SGLT2, on which SGLT2 inhibitors work just directly. Therefore, we need to distinguish each type of cell or each part of the kidney in discussing the SGLT2 inhibitor’s effect on the kidney.

In the kidney, autophagy is reported to play an important role especially in the proximal tubular cells and the podocytes. Proximal tubular cells are enriched in mitochondria, supplying energies needed for reabsorption of glucose and amino acids in primary urine. Damaged mitochondria cause oxidative stress and injure proximal tubular cells, so the clearance of damaged mitochondria by autophagy (mitophagy) is important. In fact, damaged mitochondria and degenerated proteins are degraded by autophagy in the acute kidney injury of the I/R model (Kimura et al., 2011). In addition, autophagy of proximal tubular cells acts in the degradation of reabsorbed albumin from primary urine (Yamahara et al., 2013), in lipid metabolism (Yamamoto et al., 2017), and in the protective response to DNA damage by cisplatin (Takahashi et al., 2012). Podocytes exist in the outer layer of the glomerular capillary lumen, preventing the translocation of proteins and large molecules from the capillary lumen into the urinary space. The podocyte is one of the terminally differentiated cells and poor at regeneration by cell division, so autophagy plays an important role in the response to the damaging stress of the podocyte. Therefore, the autophagic activity of podocytes is kept comparably high under normal conditions (Suzuki et al., 2019; Bork et al., 2020). In fact, podocyte-specific autophagy-deficient mice show the exacerbation of the urinary protein level (Mizushima et al., 2004; Hartleben et al., 2010).

In DKD, autophagy stagnation occurs in proximal tubular cells and podocytes, and SGLT2 inhibitors are reported to ameliorate both. SGLT2 inhibitors are reported to suppress mTORC1 and enhance the autophagy of damaged mitochondria in the proximal tubular cells of diabetic and obesity mice (Lee et al., 2019; Fukushima et al., 2020). mTORC1 is suppressed by SGLT2 inhibitors *via* the decrease in glucose and insulin and *via* the increase in ketone body production in proximal tubular cells (Tomita et al., 2020). SGLT2 inhibitors are also reported to improve the autophagy flux of podocytes by the suppression of BCL2 and activation of Beclin1 in diabetic mice (Korbut et al., 2020). Besides, SGLT2 inhibitors upregulate SIRT1 (Umino et al., 2018) and HIF-2α (Chen et al., 2011; Chen et al., 2012) in DKD, which would also contribute to ameliorate the autophagy stagnation. These therapeutic effects would be due to the systemic fasting–like state by SGLT2 inhibitors, but SGLT2 inhibitors would affect locally and directly on proximal tubular cells, for SGLT2 is abundantly expressed in proximal tubular cells, and it becomes over-upregulated in DKD (Umino et al., 2018). *In vitro* studies feeding proximal tubular cells in high-glucose medium showed SGLT2 inhibitor’s therapeutic effects on autophagy (Lee et al., 2019; Fukushima et al., 2020), indicating SGLT2 inhibitor’s direct effect on the autophagy of proximal tubular cells. Besides, Cassis et al. (2018) reported that podocytes express SGLT2 and upregulate it in response to albumin injection, and SGLT2 inhibitors directly limit podocyte damage in proteinuric nephropathy, indicating that SGLT2 inhibitors might directly improve the autophagic deficiency in podocytes, like in proximal tubular cells, of DKD.

**FIGURE 2:**
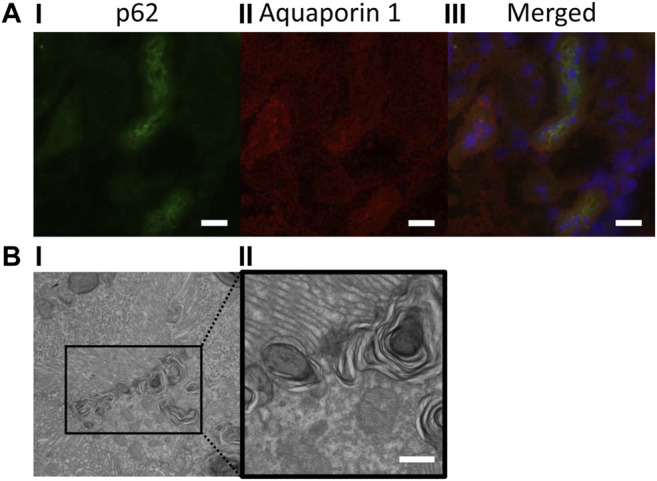
p62-positive spots and multi-lamellar bodies (MLBs) accumulated under or on the brush border of proximal tubular cells. **(A)** Immunofluorescent images (400× magnification); **(I)** p62 (green), **(II)** aquaporin-1 (red), and **(III)** merged, bars: 20 μm; **(B)** electron microscopic images (**i**: 1,500× magnification and **ii**: 6,000× magnification); MLBs in proximal tubular cells, bar: 500 nm.

In addition, the improvement of lipid metabolism by SGLT2 inhibitors would improve the degradation capacity of lysosomes, which would contribute to the amelioration of autophagy flux (Fukushima et al., 2020). In the clearance of damaged organelles by the autophagy mechanism, not only lysosomal degradation but also the exocytosis of degradants plays an important role, that is, the secretory autophagy (Kimura et al., 2017; Buratta et al., 2020). In the secretory autophagy, degradants in autophagosomes are discharged by their exocytosis or discharged after having fused with endosomes and/or lysosomes. In proximal tubular cells, autolysosomes that contain phospholipid forms multi-lamellar bodies (MLBs), which can be confirmed by the electron microscope, and the MLBs are reported to appear in urine (Rampanelli et al., 2018). Our previous study confirmed the MLBs and the autophagic marker p62-positive spots in the proximal tubular cells of obesity mice, and some of them were accumulated under or on the brush border (Figure 2), indicating the MLBs would be secreted in urine. As there are some reports that in some timings and conditions SGLT2 inhibitors increase the amount of lysosomal enzymes in urine (van Meer et al., 2016; Nunoi et al., 2018), it would be of interest to examine the relation of SGLT2 inhibitors and the secretory autophagy. Thus, SGLT2 inhibitors improve the renal autophagy deficiency *via* various mechanisms.

### Other Organs

In addition to the kidney, SGLT2 inhibitors also improve the autophagy deficiency of the systemic organs which do not express SGLT2 (Table 1). Among them, the heart is well-studied to demonstrate the therapeutic effects of SGLT2 inhibitors comparable to the kidney. Inhibition of Na^+^ and glucose reabsorption by SGLT2 inhibitors brings cardioprotective effects *via* hemodynamic and metabolic mechanisms. The increase in Na^+^ excretion into urine reduces cardiac filling pressures and causes neurohormonal changes, resulting in cardioprotective effects (Packer, 2020c). Furthermore, the increase in glucose excretion activates SIRT1 signaling and increases ketogenesis, resulting in cardioprotective effects in DKD (Cowie and Fisher, 2020). These effects are basically considered to be due to the inhibition of SGLT2 on proximal tubular cells, but some studies suggest that SGLT2 inhibitors work directly on myocardial cells. In an *in vitro* study, SGLT2 inhibitors decrease the intracellular concentration of Na^+^ and pH of myocardial cells, indicating that SGLT2 inhibitors directly inhibit Na^+^/H^+^ exchanger (NHE) 1 (Baartscheer et al., 2017; Uthman et al., 2018). Besides, *in silico* analysis indicates the SGLT2 inhibitor’s binding affinities to the structural model of NHE1 (Jiang et al., 2021). More studies are needed to elucidate the way SGLT2 inhibitors work on myocardial cells.

SGLT2 inhibitors are reported to improve the autophagy deficiency of cardiomyocytes. Cardiomyocytes are the terminally differentiated cells and rarely perform cell division, except for in specific situations (Hesse et al., 2018), so autophagy plays an important role in maintaining homeostasis of cardiomyocytes. In diabetes, autophagy deficiency causes cardiomyopathy, and SGLT2 inhibitors are reported to mitigate the autophagy deficiency, resulting in cardioprotective effect (Aragon-Herrera et al., 2019; Zhang et al., 2020). Several pathways are reported in improving the myocardial cell’s autophagy deficiency of diabetes, such as the activation of AMPK (Aragon-Herrera et al., 2019) and through the microRNA-30d/KLF9/VEGFA pathway (Zhang et al., 2020). SGLT2 inhibitors are also reported to suppress mTORC1 formation (Ren et al., 2021) and activate SIRT1 (Packer, 2020b) and HIF-2α (Packer, 2020a) of myocardial cells, which could contribute to the improvement of autophagy.

Most studies on SGLT2 inhibitor’s effects on autophagy suggest the activation of stagnated autophagy flux; however, there is a report suggesting that SGLT2 inhibitors ameliorate overactivated autophagy in cardiomyocytes. Jiang et al. (2021) reported that SGLT2 inhibitors downregulated myocardial autophagy flux of myocardial infarction mice and suppressed autophagic cell death (ACD) of cardiomyocytes—autosis, resulting in myocardial protection. ACD is one of the programmed cell deaths induced by autophagy reported to occur such as in the development process of *Drosophila* (Tracy and Baehrecke, 2013) and when apoptosis is suppressed by double knockout of Bax/Bak in mammalian cells (Shimizu et al., 2004). Autosis is regarded as one of ACDs that are reported to occur in the reperfusion phase of the I/R mouse model of cardiomyocytes (Matsui et al., 2007). Na/K-ATPase activation and following activation of Beclin1 is considered essential for autosis (Liu et al., 2013), and the Beclin1 activation is reported to be suppressed by SGLT2 inhibitors *via* inhibition of myocardial NHE1 in the I/R mouse model (Jiang et al., 2021). The concept and definition of the autosis call for more research and discussion; however, it is interesting that SGLT2 inhibitors downregulated autophagy flux, or at least, did not hyperactivate autophagy flux in the reperfusion phase of the I/R myocardial model. Besides, there is one report which suggests that SGLT2 inhibitors suppressed doxorubicin-induced autophagy in HepG2 cells *via* promoting ULK1 serine 757 phosphorylation (Zhong et al., 2020). These indicate that SGLT2 inhibitors could behave as a “regulator,” not just as enhancer, of autophagy activity. Thus, SGLT2 inhibitors improve autophagy deficiencies of the systemic organs which do not express SGLT2 *via* various mechanisms.

## Discussion

As we discussed, SGLT2 inhibitors affect the autophagy flux of multiple organs and diseases *via* various mechanisms. Considering the importance of autophagy in maintaining the cellular homeostasis, autophagy-targeting therapy would be of great significance in clinical use. In fact, some medicines working on autophagy-modulating molecules are beneficial in clinical settings, although many of these medicines also have autophagy-independent therapeutic effects, and it is hard to distinguish them. Metformin, an antidiabetic drug of AMPK activator, improves autophagy deficiency and has protective effects on multiple organs (Xie et al., 2011). Rapamycin, an mTOR inhibitor used in autoimmune diseases and cancers also improves autophagy deficiency (Su et al., 2018) and has renal protective effects in DKD (Yang et al., 2007; Mori et al., 2009).

One of the characteristics of SGLT2 inhibitor’s effects on autophagy is the “indirectivity”; SGLT2 inhibitors are not the agonist or antagonist of the specific autophagy-modulating molecules but affect the autophagy flux *via* producing the fasting-like state due to glucose excretion into urine. This indirectivity would enable SGLT2 inhibitors to affect the autophagy-regulating signaling systematically, such as mTORC1, SIRT1, and HIFs. For example, metformin could also activate SIRT1 (Song et al., 2015; Ren et al., 2020), but rather inhibits HIF-1α and HIF-2α (Treins et al., 2006; Yang et al., 2018), as expected from AMPK activation (Figure 1). Considering that autophagy consists of multiple steps and each of them is controlled by various signaling pathways, the systematic effect of SGLT2 inhibitors on autophagy would be beneficial. Of course, SGLT2 inhibitor’s effects on various signaling also suggest the risk of side effects. It is well-known that euglycemic diabetic ketoacidosis is the fatal side effect of SGLT2 inhibitors, which might occur on a sick day (Ogawa and Sakaguchi, 2016). Like the other antidiabetic drugs, SGLT2 inhibitors need to be used carefully depending on the patient’s general conditions.

There is one concern about the autophagy-targeting therapy, that is, the activation of autophagy sometimes could induce bad effects. In the previous section, we discussed the myocardial cell death by the excessive autophagy (autosis) in the I/R model. In addition, in the aging mouse model, the decrease in rubicon1 in the adipocyte causes the excessive autophagy, while accumulation of rubicon1 in the hepatocytes causes the stagnation of autophagy at the same time, and the former autophagy deficiency causes impaired glucose tolerance and weight loss (Yamamuro et al., 2020). It is not elucidated whether excessive autophagy occurs in other organs including the kidney; however, these indicate that simply activating therapies of autophagy could be the “double-edged sword” in some situations. Although there is no evidence at present, but theoretically, there might be a risk that SGLT2 inhibitors promote the excessive autophagy, for the autophagy activation in adipocytes as the result of the decrease in rubicon1 occurs in starvation to supply the whole body with energies from lipids (Yamamuro et al., 2020), and SGLT2 inhibitors just promote the fasting-like state. However, such an excessive autophagy would be preventive in the clinical use of SGLT2 inhibitors when the patient is supplied with enough energy and free from starvation. In contrast, in the autophagy-activating therapy by targeting the single autophagy-modulating molecule, it would be more difficult to cope with both the overactivation and stagnation of autophagy at the same time. As we discussed, SGLT2 inhibitors not only activate but also suppress the autophagy flux depending on the situation, indicating that SGLT2 inhibitors can work on the autophagy in a way that does not disturb the self-regulation of autophagy. This might be due to the “indirectivity” and “systematicity” of SGLT2 inhibitor’s effect on the autophagy, enabling that the homeostasis of the whole body can determine the appropriate activity of autophagy in each organ and situation. Thus, the characteristic of SGTL2 inhibitors as the “regulator” of autophagy would be beneficial in the treatment of the autophagy deficiency.

## Conclusion

Autophagy improvement by SGLT2 inhibitors would be of great significance considering the importance of autophagy in maintaining the homeostasis of the cells and organs. Autophagy-targeting therapy is difficult because the autophagy is controlled by various signaling pathways in each step, and the appropriate activity of the autophagy is different depending on the organs and diseases. SGLT2 inhibitors affect the autophagy indirectly and systematically, which enables to control various autophagy-regulating signaling pathways. Besides, in the use of SGLT2 inhibitors, it is not the drugs themselves but the homeostasis of the whole body that determines the activity of autophagy in each organ and condition, which might prevent the inappropriate activation of autophagy. More studies are needed to evaluate the SGLT2 inhibitor’s effects on the autophagy in clinical settings.

## Glossary

ACD: Autophagic cell death
ADPKD: Autosomal dominant polycystic kidney disease
AGEs: Advanced glycation end products
Akt: Protein kinase B
AMPK: AMP-activated protein kinase
ATG: Autophagy-related gene
BCL2: B-cell lymphoma 2
BNIP3: BCL2 interacting protein 3
DKD: Diabetic kidney disease
DM: Diabetes mellitus
DOX: Doxorubicin
FOXO3a: Forkhead box O3a
HIF: Hypoxia-inducible factor
HRE: Hypoxia-responsive elements
I/R: Ischemia–reperfusion
KLF9: Kruppel-like factor 9
LKB1: Liver kinase B1
MLBs: Multi-lamellar bodies
mTOR: Mammalian target of rapamycin
mTORC1: Mammalian target of rapamycin complex 1
NAD: Nicotinamide adenine dinucleotide
NAFLD: Non-alcoholic fatty liver disease
NHE1: Na^+^/H^+^ exchanger 1
PDK: Phosphoinositide-dependent kinase
PHD: Prolyl hydroxylases
PI3K: Phosphatidylinositol-3 kinase
ROS: Reactive oxygen species
Rheb: Ras homolog enriched in brain
SGLT2: Sodium–glucose cotransporter 2
SIRT1: Siruin 1
SNT: Sunitinib
SREBP: Sterol regulatory element–binding protein
TSC: Tuberous sclerosis complex
ULK1: Unc-51–like autophagy-activating kinase 1
VEGFA: Vascular endothelial growth factor A

## References

[B1] Arab H. H. Al-Shorbagy M. Y. Saad M. A. (2021). Activation of Autophagy and Suppression of Apoptosis by Dapagliflozin Attenuates Experimental Inflammatory Bowel Disease in Rats: Targeting AMPK/mTOR, HMGB1/RAGE and Nrf2/HO-1 Pathways. Chem. Biol. Interact. 335, 109368. 10.1016/j.cbi.2021.109368 33412153

[B2] Aragón-Herrera A. Feijóo-Bandín S. Otero Santiago M. Barral L. Campos-Toimil M. Gil-Longo J. (2019). Empagliflozin Reduces the Levels of CD36 and Cardiotoxic Lipids while Improving Autophagy in the Hearts of Zucker Diabetic Fatty Rats. Biochem. Pharmacol. 170, 113677. 10.1016/j.bcp.2019.113677 31647926

[B3] Baartscheer A. Schumacher C. A. Wüst R. C. Fiolet J. W. Stienen G. J. Coronel R. (2017). Empagliflozin Decreases Myocardial Cytoplasmic Na+ through Inhibition of the Cardiac Na+/H+ Exchanger in Rats and Rabbits. Diabetologia 60 (3), 568–573. 10.1007/s00125-016-4134-x 27752710 PMC6518059

[B4] Ban J. J. Ruthenborg R. J. Cho K. W. Kim J. W. (2014). Regulation of Obesity and Insulin Resistance by Hypoxia-Inducible Factors. Hypoxia 2, 171–183. 10.2147/HP.S68771 27774475 PMC5045065

[B5] Bellot G. Garcia-Medina R. Gounon P. Chiche J. Roux D. Pouysségur J. (2009). Hypoxia-Induced Autophagy Is Mediated through Hypoxia-Inducible Factor Induction of BNIP3 and BNIP3L via Their BH3 Domains. Mol. Cell Biol. 29 (10), 2570–2581. 10.1128/MCB.00166-09 19273585 PMC2682037

[B6] Bessho R. Takiyama Y. Takiyama T. Kitsunai H. Takeda Y. Sakagami H. (2019). Hypoxia-inducible Factor-1α is the Therapeutic Target of the SGLT2 Inhibitor for Diabetic Nephropathy. Sci. Rep. 9 (1), 14754. 10.1038/s41598-019-51343-1 31611596 PMC6791873

[B7] Bondeva T. Heinzig J. Ruhe C. Wolf G. (2013). Advanced Glycated End-Products Affect HIF-Transcriptional Activity in Renal Cells. Mol. Endocrinol. 27 (11), 1918–1933. 10.1210/me.2013-1036 24030251 PMC5427833

[B8] Bork T. Liang W. Yamahara K. Lee P. Tian Z. Liu S. (2020). Podocytes Maintain High Basal Levels of Autophagy Independent of Mtor Signaling. Autophagy 16 (11), 1932–1948. 10.1080/15548627.2019.1705007 31865844 PMC7595647

[B9] Brugarolas J. Lei K. Hurley R. L. Manning B. D. Reiling J. H. Hafen E. (2004). Regulation of mTOR Function in Response to Hypoxia by REDD1 and the TSC1/TSC2 Tumor Suppressor Complex. Genes Dev. 18 (23), 2893–2904. 10.1101/gad.1256804 15545625 PMC534650

[B10] Buratta S. Tancini B. Sagini K. Delo F. Chiaradia E. Urbanelli L. (2020). Lysosomal Exocytosis, Exosome Release and Secretory Autophagy: The Autophagic- and Endo-Lysosomal Systems Go Extracellular. Int. J. Mol. Sci. 21 (7). 10.3390/ijms21072576 PMC7178086 32276321

[B11] Cantó C. Gerhart-Hines Z. Feige J. N. Lagouge M. Noriega L. Milne J. C. (2009). AMPK Regulates Energy Expenditure by Modulating NAD+ Metabolism and SIRT1 Activity. Nature 458 (7241), 1056–1060. 10.1038/nature07813 19262508 PMC3616311

[B12] Cassis P. Locatelli M. Cerullo D. Corna D. Buelli S. Zanchi C. (2018). SGLT2 Inhibitor Dapagliflozin Limits Podocyte Damage in Proteinuric Nondiabetic Nephropathy. JCI Insight 3 (15). 10.1172/jci.insight.98720 PMC6129124 30089717

[B13] Chen R. Dioum E. M. Hogg R. T. Gerard R. D. Garcia J. A. (2011). Hypoxia Increases Sirtuin 1 Expression in a Hypoxia-Inducible Factor-dependent Manner. J. Biol. Chem. 286 (16), 13869–13878. 10.1074/jbc.M110.175414 21345792 PMC3077588

[B14] Chen R. Jiang T. She Y. Xu J. Li C. Zhou S. (2017). Effects of Cobalt Chloride, a Hypoxia-Mimetic Agent, on Autophagy and Atrophy in Skeletal C2C12 Myotubes. Biomed. Res. Int. 2017, 7097580. 10.1155/2017/7097580 28706950 PMC5494548

[B15] Chen R. Xu M. Hogg R. T. Li J. Little B. Gerard R. D. (2012). The Acetylase/Deacetylase Couple CREB-Binding protein/Sirtuin 1 Controls Hypoxia-Inducible Factor 2 Signaling. J. Biol. Chem. 287 (36), 30800–30811. 10.1074/jbc.M111.244780 22807441 PMC3436323

[B16] Cherney D. Z. Perkins B. A. Soleymanlou N. Maione M. Lai V. Lee A. (2014). Renal Hemodynamic Effect of Sodium-Glucose Cotransporter 2 Inhibition in Patients with Type 1 Diabetes Mellitus. Circulation 129 (5), 587–597. 10.1161/CIRCULATIONAHA.113.005081 24334175

[B17] Cowie M. R. Fisher M. (2020). SGLT2 Inhibitors: Mechanisms of Cardiovascular Benefit beyond Glycaemic Control. Nat. Rev. Cardiol. 17 (12), 761–772. 10.1038/s41569-020-0406-8 32665641

[B18] DeFronzo R. A. Reeves W. B. Awad A. S. (2021). Pathophysiology of Diabetic Kidney Disease: Impact of SGLT2 Inhibitors. Nat. Rev. Nephrol. 17 (5), 319–334. 10.1038/s41581-021-00393-8 33547417

[B19] Dibble C. C. Cantley L. C. (2015). Regulation of mTORC1 by PI3K Signaling. Trends Cel Biol 25 (9), 545–555. 10.1016/j.tcb.2015.06.002 PMC4734635 26159692

[B20] Eckardt K. U. Kurtz A. (2005). Regulation of Erythropoietin Production. Eur. J. Clin. Invest. 35 (Suppl. 3), 13–19. 10.1111/j.1365-2362.2005.01525.x 16281953

[B21] El-Sahar A. E. Rastanawi A. A. El-Yamany M. F. Saad M. A. (2020). Dapagliflozin Improves Behavioral Dysfunction of Huntington's Disease in Rats via Inhibiting Apoptosis-Related Glycolysis. Life Sci. 257, 118076. 10.1016/j.lfs.2020.118076 32659371

[B22] Esterline R. Oscarsson J. Burns J. (2020). A Role for Sodium Glucose Cotransporter 2 Inhibitors (SGLT2is) in the Treatment of Alzheimer's Disease. Int. Rev. Neurobiol. 155, 113–140. 10.1016/bs.irn.2020.03.018 32854852

[B23] Esterline R. L. Vaag A. Oscarsson J. Vora J. (2018). Mechanisms in Endocrinology: SGLT2 Inhibitors: Clinical Benefits by Restoration of normal Diurnal Metabolism. Eur. J. Endocrinol. 178 (4), R113–R125. 10.1530/EJE-17-0832 29371333

[B24] Fang E. F. Scheibye-Knudsen M. Brace L. E. Kassahun H. SenGupta T. Nilsen H. (2014). Defective Mitophagy in XPA via PARP-1 Hyperactivation and NAD(+)/SIRT1 Reduction. Cell 157 (4), 882–896. 10.1016/j.cell.2014.03.026 24813611 PMC4625837

[B25] Fukushima K. Kitamura S. Tsuji K. Sang Y. Wada J. (2020). Sodium Glucose Co-transporter 2 Inhibitor Ameliorates Autophagic Flux Impairment on Renal Proximal Tubular Cells in Obesity Mice. Int. J. Mol. Sci. 21 (11). 10.3390/ijms21114054 PMC7312919 32517059

[B26] Ghosh H. S. McBurney M. Robbins P. D. (2010). SIRT1 Negatively Regulates the Mammalian Target of Rapamycin. PLoS One 5 (2), e9199. 10.1371/journal.pone.0009199 20169165 PMC2821410

[B27] Gillum M. P. Erion D. M. Shulman G. I. (2011). Sirtuin-1 Regulation of Mammalian Metabolism. Trends Mol. Med. 17 (1), 8–13. 10.1016/j.molmed.2010.09.005 20971038 PMC3123438

[B28] Hartleben B. Gödel M. Meyer-Schwesinger C. Liu S. Ulrich T. Köbler S. (2010). Autophagy Influences Glomerular Disease Susceptibility and Maintains Podocyte Homeostasis in Aging Mice. J. Clin. Invest. 120 (4), 1084–1096. 10.1172/JCI39492 20200449 PMC2846040

[B29] Hasegawa K. Wakino S. Simic P. Sakamaki Y. Minakuchi H. Fujimura K. (2013). Renal Tubular Sirt1 Attenuates Diabetic Albuminuria by Epigenetically Suppressing Claudin-1 Overexpression in Podocytes. Nat. Med. 19 (11), 1496–1504. 10.1038/nm.3363 24141423 PMC4041199

[B30] Hesse M. Welz A. Fleischmann B. K. (2018). Heart Regeneration and the Cardiomyocyte Cell Cycle. Pflugers Arch. 470 (2), 241–248. 10.1007/s00424-017-2061-4 28849267 PMC5780532

[B31] Hirota K. (2021). HIF-α Prolyl Hydroxylase Inhibitors and Their Implications for Biomedicine: A Comprehensive Review. Biomedicines 9 (5). 10.3390/biomedicines9050468 PMC8146675 33923349

[B32] Iside C. Scafuro M. Nebbioso A. Altucci L. (2020). SIRT1 Activation by Natural Phytochemicals: An Overview. Front. Pharmacol. 11, 1225. 10.3389/fphar.2020.01225 32848804 PMC7426493

[B33] Isoe T. Makino Y. Mizumoto K. Sakagami H. Fujita Y. Honjo J. (2010). High Glucose Activates HIF-1-Mediated Signal Transduction in Glomerular Mesangial Cells Through a Carbohydrate Response Element Binding Protein. Kidney Int. 78 (1), 48–59. 10.1038/ki.2010.99 20375990

[B34] Jaikumkao K. Promsan S. Thongnak L. Swe M. T. Tapanya M. Htun K. T. (2021). Dapagliflozin Ameliorates Pancreatic Injury and Activates Kidney Autophagy by Modulating the AMPK/mTOR Signaling Pathway in Obese Rats. J. Cel Physiol 236 (9), 6424–6440. 10.1002/jcp.30316 33559163

[B35] Jewell J. L. Guan K. L. (2013). Nutrient Signaling to mTOR and Cell Growth. Trends Biochem. Sci. 38 (5), 233–242. 10.1016/j.tibs.2013.01.004 23465396 PMC3634910

[B36] Jiang K. Xu Y. Wang D. Chen F. Tu Z. Qian J. (2021). Cardioprotective Mechanism of SGLT2 Inhibitor against Myocardial Infarction Is through Reduction of Autosis. Protein Cell. 10.1007/s13238-020-00809-4 PMC9008115 33417139

[B37] Jürgensen J. S. Rosenberger C. Wiesener M. S. Warnecke C. Hörstrup J. H. Gräfe M. (2004). Persistent Induction of HIF-1alpha and -2alpha in Cardiomyocytes and Stromal Cells of Ischemic Myocardium. FASEB J. 18 (12), 1415–1417. 10.1096/fj.04-1605fje 15247145

[B38] Kamezaki M. Kusaba T. Komaki K. Fushimura Y. Watanabe N. Ikeda K. (2018). Comprehensive Renoprotective Effects of Ipragliflozin on Early Diabetic Nephropathy in Mice. Sci. Rep. 8 (1), 4029. 10.1038/s41598-018-22229-5 29507299 PMC5838225

[B39] Kidokoro K. Cherney D. Z. I. Bozovic A. Nagasu H. Satoh M. Kanda E. (2019). Evaluation of Glomerular Hemodynamic Function by Empagliflozin in Diabetic Mice Using *In Vivo* Imaging. Circulation 140 (4), 303–315. 10.1161/CIRCULATIONAHA.118.037418 30773020

[B40] Kim S. Jo C. H. Kim G. H. (2019). Effects of Empagliflozin on Nondiabetic Salt-Sensitive Hypertension in Uninephrectomized Rats. Hypertens. Res. 42 (12), 1905–1915. 10.1038/s41440-019-0326-3 31537914 PMC8075936

[B41] Kimura T. Jia J. Kumar S. Choi S. W. Gu Y. Mudd M. (2017). Dedicated SNAREs and Specialized TRIM Cargo Receptors Mediate Secretory Autophagy. EMBO J. 36 (1), 42–60. 10.15252/embj.201695081 27932448 PMC5210154

[B42] Kimura T. Takabatake Y. Takahashi A. Kaimori J. Y. Matsui I. Namba T. (2011). Autophagy Protects the Proximal Tubule from Degeneration and Acute Ischemic Injury. J. Am. Soc. Nephrol. 22 (5), 902–913. 10.1681/ASN.2010070705 21493778 PMC3083312

[B43] Kitada M. Ogura Y. Monno I. Koya D. (2017). Regulating Autophagy as a Therapeutic Target for Diabetic Nephropathy. Curr. Diab Rep. 17 (7), 53. 10.1007/s11892-017-0879-y 28593583

[B44] Kong K. H. Oh H. J. Lim B. J. Kim M. Han K. H. Choi Y. H. (2017). Selective Tubular Activation of Hypoxia-Inducible Factor-2α Has Dual Effects on Renal Fibrosis. Sci. Rep. 7 (1), 11351. 10.1038/s41598-017-11829-2 28900259 PMC5596020

[B45] Korbut A. I. Taskaeva I. S. Bgatova N. P. Muraleva N. A. Orlov N. B. Dashkin M. V. (2020). SGLT2 Inhibitor Empagliflozin and DPP4 Inhibitor Linagliptin Reactivate Glomerular Autophagy in db/db Mice, a Model of Type 2 Diabetes. Int. J. Mol. Sci. 21 (8). 10.3390/ijms21082987 PMC7215949 32340263

[B46] Kume S. Uzu T. Horiike K. Chin-Kanasaki M. Isshiki K. Araki S. (2010). Calorie Restriction Enhances Cell Adaptation to Hypoxia through Sirt1-dependent Mitochondrial Autophagy in Mouse Aged Kidney. J. Clin. Invest. 120 (4), 1043–1055. 10.1172/JCI41376 20335657 PMC2846062

[B47] Lee I. H. (2019). Mechanisms and Disease Implications of Sirtuin-Mediated Autophagic Regulation. Exp. Mol. Med. 51 (9), 1–11. 10.1038/s12276-019-0302-7 PMC6802627 31492861

[B48] Lee Y. H. Kim S. H. Kang J. M. Heo J. H. Kim D. J. Park S. H. (2019). Empagliflozin Attenuates Diabetic Tubulopathy by Improving Mitochondrial Fragmentation and Autophagy. Am. J. Physiol. Ren. Physiol 317 (4), F767–F780. 10.1152/ajprenal.00565.2018 31390268

[B49] Li L. Li Q. Huang W. Han Y. Tan H. An M. (2021). Dapagliflozin Alleviates Hepatic Steatosis by Restoring Autophagy via the AMPK-mTOR Pathway. Front. Pharmacol. 12, 589273. 10.3389/fphar.2021.589273 34093169 PMC8176308

[B50] Li S. Brown M. S. Goldstein J. L. (2010). Bifurcation of Insulin Signaling Pathway in Rat Liver: mTORC1 Required for Stimulation of Lipogenesis, but Not Inhibition of Gluconeogenesis. Proc. Natl. Acad. Sci. U S A. 107 (8), 3441–3446. 10.1073/pnas.0914798107 20133650 PMC2840492

[B51] Liu Y. Shoji-Kawata S. Sumpter R. M. Jr. Wei Y. Ginet V. Zhang L. (2013). Autosis Is a Na+,K+-ATPase-regulated Form of Cell Death Triggered by Autophagy-Inducing Peptides, Starvation, and Hypoxia-Ischemia. Proc. Natl. Acad. Sci. U S A. 110 (51), 20364–20371. 10.1073/pnas.1319661110 24277826 PMC3870705

[B52] Luo R. Zhang W. Zhao C. Zhang Y. Wu H. Jin J. (2015). Elevated Endothelial Hypoxia-Inducible Factor-1α Contributes to Glomerular Injury and Promotes Hypertensive Chronic Kidney Disease. Hypertension 66 (1), 75–84. 10.1161/HYPERTENSIONAHA.115.05578 25987665 PMC4752003

[B53] Matsui Y. Takagi H. Qu X. Abdellatif M. Sakoda H. Asano T. (2007). Distinct Roles of Autophagy in the Heart during Ischemia and Reperfusion: Roles of AMP-Activated Protein Kinase and Beclin 1 in Mediating Autophagy. Circ. Res. 100 (6), 914–922. 10.1161/01.RES.0000261924.76669.36 17332429

[B54] Meng Z. Liu X. Li T. Fang T. Cheng Y. Han L. (2021). The SGLT2 Inhibitor Empagliflozin Negatively Regulates IL-17/IL-23 axis-mediated Inflammatory Responses in T2DM with NAFLD via the AMPK/mTOR/autophagy Pathway. Int. Immunopharmacol. 94, 107492. 10.1016/j.intimp.2021.107492 33647823

[B55] Mitra M. S. Donthamsetty S. White B. Mehendale H. M. (2008). High Fat Diet-Fed Obese Rats Are Highly Sensitive to Doxorubicin-Induced Cardiotoxicity. Toxicol. Appl. Pharmacol. 231 (3), 413–422. 10.1016/j.taap.2008.05.006 18674790

[B56] Mizuno M. Kuno A. Yano T. Miki T. Oshima H. Sato T. (2018). Empagliflozin Normalizes the Size and Number of Mitochondria and Prevents Reduction in Mitochondrial Size after Myocardial Infarction in Diabetic Hearts. Physiol. Rep. 6 (12), e13741. 10.14814/phy2.13741 29932506 PMC6014462

[B57] Mizushima N. (2010). The Role of the Atg1/ULK1 Complex in Autophagy Regulation. Curr. Opin. Cel Biol 22 (2), 132–139. 10.1016/j.ceb.2009.12.004 20056399

[B58] Mizushima N. Yamamoto A. Matsui M. Yoshimori T. Ohsumi Y. (2004). *In Vivo* analysis of Autophagy in Response to Nutrient Starvation Using Transgenic Mice Expressing a Fluorescent Autophagosome Marker. Mol. Biol. Cell 15 (3), 1101–1111. 10.1091/mbc.e03-09-0704 14699058 PMC363084

[B59] Mori H. Inoki K. Masutani K. Wakabayashi Y. Komai K. Nakagawa R. (2009). The mTOR Pathway Is Highly Activated in Diabetic Nephropathy and Rapamycin Has a strong Therapeutic Potential. Biochem. Biophys. Res. Commun. 384 (4), 471–475. 10.1016/j.bbrc.2009.04.136 19422788

[B60] Nasiri-Ansari N. Nikolopoulou C. Papoutsi K. Kyrou I. Mantzoros C. S. Kyriakopoulos G. (2021). Empagliflozin Attenuates Non-alcoholic Fatty Liver Disease (NAFLD) in High Fat Diet Fed ApoE(-/-) Mice by Activating Autophagy and Reducing ER Stress and Apoptosis. Int. J. Mol. Sci. 22 (2). 10.3390/ijms22020818 PMC7829901 33467546

[B61] Nowak K. L. Hopp K. (2020). Metabolic Reprogramming in Autosomal Dominant Polycystic Kidney Disease: Evidence and Therapeutic Potential. Clin. J. Am. Soc. Nephrol. 15 (4), 577–584. 10.2215/CJN.13291019 32086281 PMC7133124

[B62] Nunoi K. Sato Y. Kaku K. Yoshida A. Suganami H. (2018). Effects of Sodium-Glucose Cotransporter 2 Inhibitor, Tofogliflozin, on the Indices of Renal Tubular Function in Patients with Type 2 Diabetes. Endocrinol. Diabetes Metab. 1 (2), e00015. 10.1002/edm2.15 30815551 PMC6354802

[B63] Ogawa W. Sakaguchi K. (2016). Euglycemic Diabetic Ketoacidosis Induced by SGLT2 Inhibitors: Possible Mechanism and Contributing Factors. J. Diabetes Investig. 7 (2), 135–138. 10.1111/jdi.12401 PMC4773669 27042263

[B64] Osataphan S. Macchi C. Singhal G. Chimene-Weiss J. Sales V. Kozuka C. (2019). SGLT2 Inhibition Reprograms Systemic Metabolism via FGF21-dependent and -independent Mechanisms. JCI Insight 4 (5). 10.1172/jci.insight.123130 PMC6483601 30843877

[B65] Packer M. (2020a). Autophagy Stimulation and Intracellular Sodium Reduction as Mediators of the Cardioprotective Effect of Sodium-Glucose Cotransporter 2 Inhibitors. Eur. J. Heart Fail. 22 (4), 618–628. 10.1002/ejhf.1732 32037659

[B66] Packer M. (2020b). Cardioprotective Effects of Sirtuin-1 and its Downstream Effectors: Potential Role in Mediating the Heart Failure Benefits of SGLT2 (Sodium-Glucose Cotransporter 2) Inhibitors. Circ. Heart Fail. 13 (9), e007197. 10.1161/CIRCHEARTFAILURE.120.007197 32894987

[B67] Packer M. (2020c). Molecular, Cellular, and Clinical Evidence that Sodium-Glucose Cotransporter 2 Inhibitors Act as Neurohormonal Antagonists when Used for the Treatment of Chronic Heart Failure. J. Am. Heart Assoc. 9 (16), e016270. 10.1161/JAHA.120.016270 32791029 PMC7660825

[B68] Packer M. (2020d). Role of Deranged Energy Deprivation Signaling in the Pathogenesis of Cardiac and Renal Disease in States of Perceived Nutrient Overabundance. Circulation 141 (25), 2095–2105. 10.1161/CIRCULATIONAHA.119.045561 32164457

[B69] Packer M. (2020e). Role of Impaired Nutrient and Oxygen Deprivation Signaling and Deficient Autophagic Flux in Diabetic CKD Development: Implications for Understanding the Effects of Sodium-Glucose Cotransporter 2-Inhibitors. J. Am. Soc. Nephrol. 31 (5), 907–919. 10.1681/ASN.2020010010 32276962 PMC7217421

[B70] Perkovic V. Jardine M. J. Neal B. Bompoint S. Heerspink H. J. L. Charytan D. M. (2019). Canagliflozin and Renal Outcomes in Type 2 Diabetes and Nephropathy. N. Engl. J. Med. 380 (24), 2295–2306. 10.1056/NEJMoa1811744 30990260

[B71] Price N. L. Gomes A. P. Ling A. J. Duarte F. V. Martin-Montalvo A. North B. J. (2012). SIRT1 Is Required for AMPK Activation and the Beneficial Effects of Resveratrol on Mitochondrial Function. Cell Metab. 15 (5), 675–690. 10.1016/j.cmet.2012.04.003 22560220 PMC3545644

[B72] Rampanelli E. Ochodnicky P. Vissers J. P. Butter L. M. Claessen N. Calcagni A. (2018). Excessive Dietary Lipid Intake Provokes an Acquired Form of Lysosomal Lipid Storage Disease in the Kidney. J. Pathol. 246 (4), 470–484. 10.1002/path.5150 30073645

[B73] Rankin E. B. Biju M. P. Liu Q. Unger T. L. Rha J. Johnson R. S. (2007). Hypoxia-inducible Factor-2 (HIF-2) Regulates Hepatic Erythropoietin *In Vivo* . J. Clin. Invest. 117 (4), 1068–1077. 10.1172/JCI30117 17404621 PMC1838939

[B74] Ren C. Sun K. Zhang Y. Hu Y. Hu B. Zhao J. (2021). Sodium-Glucose CoTransporter-2 Inhibitor Empagliflozin Ameliorates Sunitinib-Induced Cardiac Dysfunction via Regulation of AMPK-mTOR Signaling Pathway-Mediated Autophagy. Front. Pharmacol. 12, 664181. 10.3389/fphar.2021.664181 33995090 PMC8116890

[B75] Ren H. Shao Y. Wu C. Ma X. Lv C. Wang Q. (2020). Metformin Alleviates Oxidative Stress and Enhances Autophagy in Diabetic Kidney Disease via AMPK/SIRT1-FoxO1 Pathway. Mol. Cel Endocrinol 500, 110628. 10.1016/j.mce.2019.110628 31647955

[B76] Rosenberger C. Mandriota S. Jürgensen J. S. Wiesener M. S. Hörstrup J. H. Frei U. (2002). Expression of Hypoxia-Inducible Factor-1alpha and -2alpha in Hypoxic and Ischemic Rat Kidneys. J. Am. Soc. Nephrol. 13 (7), 1721–1732. 10.1097/01.asn.0000017223.49823.2a 12089367

[B77] Schönenberger M. J. Kovacs W. J. (2015). Hypoxia Signaling Pathways: Modulators of Oxygen-Related Organelles. Front. Cell Dev. Biol. 3, 42. 10.3389/fcell.2015.00042 26258123 PMC4508581

[B78] Shimizu S. Kanaseki T. Mizushima N. Mizuta T. Arakawa-Kobayashi S. Thompson C. B. (2004). Role of Bcl-2 Family Proteins in a Non-apoptotic Programmed Cell Death Dependent on Autophagy Genes. Nat. Cell Biol. 6 (12), 1221–1228. 10.1038/ncb1192 15558033

[B79] Song Y. M. Lee Y. H. Kim J. W. Ham D. S. Kang E. S. Cha B. S. (2015). Metformin Alleviates Hepatosteatosis by Restoring SIRT1-Mediated Autophagy Induction via an AMP-Activated Protein Kinase-independent Pathway. Autophagy 11 (1), 46–59. 10.4161/15548627.2014.984271 25484077 PMC4502778

[B80] Su Y. Lu J. Gong P. Chen X. Liang C. Zhang J. (2018). Rapamycin Induces Autophagy to Alleviate Acute Kidney Injury Following Cerebral Ischemia and Reperfusion via the mTORC1/ATG13/ULK1 Signaling Pathway. Mol. Med. Rep. 18 (6), 5445–5454. 10.3892/mmr.2018.9586 30365078 PMC6236225

[B81] Sugizaki T. Zhu S. Guo G. Matsumoto A. Zhao J. Endo M. (2017). Treatment of Diabetic Mice with the SGLT2 Inhibitor TA-1887 Antagonizes Diabetic Cachexia and Decreases Mortality. NPJ Aging Mech. Dis. 3, 12. 10.1038/s41514-017-0012-0 28900540 PMC5591191

[B82] Suzuki C. Tanida I. Oliva Trejo J. A. Kakuta S. Uchiyama Y. (2019). Autophagy Deficiency in Renal Proximal Tubular Cells Leads to an Increase in Cellular Injury and Apoptosis under Normal Fed Conditions. Int. J. Mol. Sci. 21 (1). 10.3390/ijms21010155 PMC6982095 31881660

[B83] Takagaki Y. Lee S. M. Dongqing Z. Kitada M. Kanasaki K. Koya D. (2020). Endothelial Autophagy Deficiency Induces IL6 - Dependent Endothelial Mesenchymal Transition and Organ Fibrosis. Autophagy 16 (10), 1905–1914. 10.1080/15548627.2020.1713641 31965901 PMC8386622

[B84] Takagi S. Li J. Takagaki Y. Kitada M. Nitta K. Takasu T. (2018). Ipragliflozin Improves Mitochondrial Abnormalities in Renal Tubules Induced by a High-Fat Diet. J. Diabetes Investig. 9 (5), 1025–1032. 10.1111/jdi.12802 PMC6123054 29352520

[B85] Takahashi A. Kimura T. Takabatake Y. Namba T. Kaimori J. Kitamura H. (2012). Autophagy Guards against Cisplatin-Induced Acute Kidney Injury. Am. J. Pathol. 180 (2), 517–525. 10.1016/j.ajpath.2011.11.001 22265049

[B86] Tanaka S. Sugiura Y. Saito H. Sugahara M. Higashijima Y. Yamaguchi J. (2018). Sodium-glucose Cotransporter 2 Inhibition Normalizes Glucose Metabolism and Suppresses Oxidative Stress in the Kidneys of Diabetic Mice. Kidney Int. 94 (5), 912–925. 10.1016/j.kint.2018.04.025 30021702

[B87] Tanaka T. Wiesener M. Bernhardt W. Eckardt K. U. Warnecke C. (2009). The Human HIF (Hypoxia-inducible Factor)-3alpha Gene Is a HIF-1 Target Gene and May Modulate Hypoxic Gene Induction. Biochem. J. 424 (1), 143–151. 10.1042/BJ20090120 19694616

[B88] Tang B. L. (2016). Sirt1 and the Mitochondria. Mol. Cell 39 (2), 87–95. 10.14348/molcells.2016.2318 PMC4757807 26831453

[B89] Tomita I. Kume S. Sugahara S. Osawa N. Yamahara K. Yasuda-Yamahara M. (2020). SGLT2 Inhibition Mediates Protection from Diabetic Kidney Disease by Promoting Ketone Body-Induced mTORC1 Inhibition. Cell Metab. 32 (3), 404–e6. 10.1016/j.cmet.2020.06.020 32726607

[B90] Tracy K. Baehrecke E. H. (2013). The Role of Autophagy in Drosophila Metamorphosis. Curr. Top. Dev. Biol. 103, 101–125. 10.1016/B978-0-12-385979-2.00004-6 23347517 PMC3896998

[B91] Treins C. Murdaca J. Van Obberghen E. Giorgetti-Peraldi S. (2006). AMPK Activation Inhibits the Expression of HIF-1alpha Induced by Insulin and IGF-1. Biochem. Biophys. Res. Commun. 342 (4), 1197–1202. 10.1016/j.bbrc.2006.02.088 16516166

[B92] Umino H. Hasegawa K. Minakuchi H. Muraoka H. Kawaguchi T. Kanda T. (2018). High Basolateral Glucose Increases Sodium-Glucose Cotransporter 2 and Reduces Sirtuin-1 in Renal Tubules through Glucose Transporter-2 Detection. Sci. Rep. 8 (1), 6791. 10.1038/s41598-018-25054-y 29717156 PMC5931531

[B93] Uthman L. Baartscheer A. Bleijlevens B. Schumacher C. A. Fiolet J. W. T. Koeman A. (2018). Class Effects of SGLT2 Inhibitors in Mouse Cardiomyocytes and Hearts: Inhibition of Na^+^/H^+^ Exchanger, Lowering of Cytosolic Na+ and Vasodilation. Diabetologia 61 (3), 722–726. 10.1007/s00125-017-4509-7 29197997 PMC6448958

[B94] van Bommel E. J. M. Muskiet M. H. A. van Baar M. J. B. Tonneijck L. Smits M. M. Emanuel A. L. (2020). The Renal Hemodynamic Effects of the SGLT2 Inhibitor Dapagliflozin are Caused by post-glomerular Vasodilatation Rather Than Pre-glomerular Vasoconstriction in Metformin-Treated Patients with Type 2 Diabetes in the Randomized, Double-Blind RED Trial. Kidney Int. 97 (1), 202–212. 10.1016/j.kint.2019.09.013 31791665

[B95] van Meer L. Moerland M. van Dongen M. Goulouze B. de Kam M. Klaassen E. (2016). Renal Effects of Antisense-Mediated Inhibition of SGLT2. J. Pharmacol. Exp. Ther. 359 (2), 280–289. 10.1124/jpet.116.233809 27605629

[B96] Walter K. M. Schönenberger M. J. Trötzmüller M. Horn M. Elsässer H. P. Moser A. B. (2014). Hif-2α Promotes Degradation of Mammalian Peroxisomes by Selective Autophagy. Cell Metab. 20 (5), 882–897. 10.1016/j.cmet.2014.09.017 25440060

[B97] Wang C. Y. Chen C. C. Lin M. H. Su H. T. Ho M. Y. Yeh J. K. (2020). TLR9 Binding to Beclin 1 and Mitochondrial SIRT3 by a Sodium-Glucose Co-transporter 2 Inhibitor Protects the Heart from Doxorubicin Toxicity. Biology 9 (11). 10.3390/biology9110369 PMC7693736 33138323

[B98] Wang X. X. Levi J. Luo Y. Myakala K. Herman-Edelstein M. Qiu L. (2017). SGLT2 Protein Expression is Increased in Human Diabetic Nephropathy: SGLT2 Protein Inhibition Decreases Renal Lipid Accumulation, Inflammation, and the Development of Nephropathy in Diabetic mice. J. Biol. Chem. 292 (13), 5335–5348. 10.1074/jbc.M117.779520 28196866 PMC5392679

[B99] Wanner C. Inzucchi S. E. Lachin J. M. Fitchett D. von Eynatten M. Mattheus M. (2016). Empagliflozin and Progression of Kidney Disease in Type 2 Diabetes. N. Engl. J. Med. 375 (4), 323–334. 10.1056/NEJMoa1515920 27299675

[B100] Xie Z. Lau K. Eby B. Lozano P. He C. Pennington B. (2011). Improvement of Cardiac Functions by Chronic Metformin Treatment Is Associated with Enhanced Cardiac Autophagy in Diabetic OVE26 Mice. Diabetes 60 (6), 1770–1778. 10.2337/db10-0351 21562078 PMC3114402

[B101] Xu C. Wang W. Zhong J. Lei F. Xu N. Zhang Y. (2018). Canagliflozin Exerts Anti-inflammatory Effects by Inhibiting Intracellular Glucose Metabolism and Promoting Autophagy in Immune Cells. Biochem. Pharmacol. 152, 45–59. 10.1016/j.bcp.2018.03.013 29551587

[B102] Yamahara K. Kume S. Koya D. Tanaka Y. Morita Y. Chin-Kanasaki M. (2013). Obesity-mediated Autophagy Insufficiency Exacerbates Proteinuria-Induced Tubulointerstitial Lesions. J. Am. Soc. Nephrol. 24 (11), 1769–1781. 10.1681/ASN.2012111080 24092929 PMC3810079

[B103] Yamamoto T. Takabatake Y. Takahashi A. Kimura T. Namba T. Matsuda J. (2017). High-Fat Diet-Induced Lysosomal Dysfunction and Impaired Autophagic Flux Contribute to Lipotoxicity in the Kidney. J. Am. Soc. Nephrol. 28 (5), 1534–1551. 10.1681/ASN.2016070731 27932476 PMC5407727

[B104] Yamamuro T. Kawabata T. Fukuhara A. Saita S. Nakamura S. Takeshita H. (2020). Age-dependent Loss of Adipose Rubicon Promotes Metabolic Disorders via Excess Autophagy. Nat. Commun. 11 (1), 4150. 10.1038/s41467-020-17985-w 32811819 PMC7434891

[B105] Yang Q. Guo X. Yang L. (2018). Metformin Enhances the Effect of Regorafenib and Inhibits Recurrence and Metastasis of Hepatic Carcinoma After Liver Resection via Regulating Expression of Hypoxia Inducible Factors 2α (HIF-2α) and 30 kDa HIV Tat-Interacting Protein (TIP30). Med. Sci. Monit. 24, 2225–2234. 10.12659/msm.906687 29654226 PMC5912093

[B106] Yang Y. Wang J. Qin L. Shou Z. Zhao J. Wang H. (2007). Rapamycin Prevents Early Steps of the Development of Diabetic Nephropathy in Rats. Am. J. Nephrol. 27 (5), 495–502. 10.1159/000106782 17671379

[B107] Zeng H. Vaka V. R. He X. Booz G. W. Chen J. X. (2015). High-fat Diet Induces Cardiac Remodelling and Dysfunction: Assessment of the Role Played by SIRT3 Loss. J. Cel Mol Med 19 (8), 1847–1856. 10.1111/jcmm.12556 PMC4549035 25782072

[B108] Zhang W. Y. Wang J. Li A. Z. (2020). A Study of the Effects of SGLT-2 Inhibitors on Diabetic Cardiomyopathy through miR-30d/KLF9/VEGFA Pathway. Eur. Rev. Med. Pharmacol. Sci. 24 (11), 6346–6359. 10.26355/eurrev_202006_21533 32572932

[B109] Zhong J. Sun P. Xu N. Liao M. Xu C. Ding Y. (2020). Canagliflozin Inhibits P-Gp Function and Early Autophagy and Improves the Sensitivity to the Antitumor Effect of Doxorubicin. Biochem. Pharmacol. 175, 113856. 10.1016/j.bcp.2020.113856 32061772

[B110] Zhu L. Qi B. Hou D. (2019). Roles of HIF1α- and HIF2α-Regulated BNIP3 in Hypoxia-Induced Injury of Neurons. Pathol. Res. Pract. 215 (4), 822–827. 10.1016/j.prp.2019.01.022 30704780

[B111] Zinman B. Wanner C. Lachin J. M. Fitchett D. Bluhmki E. Hantel S. (2015). Empagliflozin, Cardiovascular Outcomes, and Mortality in Type 2 Diabetes. N. Engl. J. Med. 373 (22), 2117–2128. 10.1056/NEJMoa1504720 26378978

